# Ocular manifestations of COVID-19 in the pediatric age group

**DOI:** 10.1177/11206721221116210

**Published:** 2022-07-27

**Authors:** Muhannad A Alnahdi, Maan Alkharashi

**Affiliations:** 1 Department of Ophthalmology and Vision Sciences, University of Toronto, Toronto, Ontario, Canada; 2 College of Medicine, King Saud bin Abdulaziz University for Health Sciences, Riyadh, Saudi Arabia; 3 Department of Ophthalmology, King Abdulaziz Medical City, Ministry of National Guard-Health Affairs, Riyadh, Saudi Arabia; 4 Department of Ophthalmology, College of Medicine, King Saud University, Riyadh, Saudi Arabia; 5 Department of Ophthalmology, Boston Children's Hospital, Boston, Massachusetts; 6 Department of Ophthalmology, Harvard Medical School, Boston, Massachusetts

**Keywords:** Pediatric ophthlamology, Neuro-ophthalmology, Orbital disease, Ocular surface, Anterior uveitis

## Abstract

The coronavirus disease 2019 (COVID-19) is now known to be associated with several ocular manifestations. The literature thoroughly discussed those that affect adults, with a lesser focus in the pediatric age group. We aim to outline the various pediatric ocular manifestations described in the literature. The manifestations may be divided into isolated events attributed to COVID-19 or occurring in the new multisystem inflammatory syndrome in children (MIS-C), a novel entity associated by COVID-19 infection. Ocular manifestations have virtually affected all ages. They manifested in neonates, infants, children, and adolescents. Episcleritis, conjunctivitis, optic neuritis, cranial nerve palsies, retinal vein occlusion, retinal vasculitis, retinal changes, orbital myositis, orbital cellulitis were reported in the literature with this emerging viral illness. Conjunctivitis was the most common ocular manifestation in MIS-C in nearly half of the patients. Other ocular manifestations in MIS-C were anterior uveitis, corneal epitheliopathy, optic neuritis, idiopathic intracranial hypertension, and retinitis. The clinical outcome was favorable, and children regain their visual ability with minimal or no deficits in most of the cases. Further follow-up may be warranted to better understand the long-term effects and visual prognosis.

## Introduction

The World Health Organization declared coronavirus disease 2019 (COVID-19) as a pandemic in March 2020. The rapid spread of this illness across countries laid an unprecedented and excessive burden upon hospitals worldwide. The first COVID-19 associated conjunctivitis was reported in a healthcare provider who developed conjunctivitis after examining a patient who later tested positive.^1^ This shed light on the potential risk of transmitting the virus through the ocular surface, necessitating practicing full precautions including eye protection. Respiratory, systemic, and unusual presentations were described in both adult and pediatric age groups. The American College of Pediatrics reported more than four million children were infected as of July 2021.^2^ Children are less infected and tend to present with milder symptoms^3–5^. Nearly a quarter of infected children initially had no symptoms, while severe to critical cases represented around ten percent, and mortality is less than one percent in children.^2,6^

Coronaviruses are known to cause ocular alterations in murine and feline models. The known clinical features were conjunctivitis, anterior uveitis, retinitis, and optic neuritis.^7^ Prior epidemiological studies in humans did not show any ocular manifestations in Severe Acute Respiratory Syndrome Coronavirus (SARS-CoV) and Middle East Respiratory Syndrome.^7^ So, most clinical implications were derived from animal models to better understand the possible manifestations of COVID-19. COVID-19 was found to have a similar receptor-binding motif as SARS-COV, which allows the virus to infect the host cells via angiotensin-converting enzyme 2 (ACE2).^8^ This receptor is part of the renin-angiotensin system expressed in endothelial cells, and preliminary reports suggest the evidence of ACE2 expression in corneal and conjunctival cells, and maybe more in the inner epithelial layers of the eye, particularly within fibroblasts and dendritic cells.^9^

We are still uncertain about the exact prevalence of ocular manifestations in COVID-19 patients. The existing reports suggest that the rate of conjunctivitis in COVID-19 patients may reach 31% in severe cases.^10^ A systematic review reports that dry eye, foreign body sensation, redness, tearing, itchiness, eye pain were the most common ocular symptoms of patients with COVID-19.^11^ The literature shows that adult patients can have various ocular manifestations associated with COVID-19, some of the reported ones are conjunctivitis, episcleritis, corneal graft rejection, orbital cellulitis, orbital inflammatory disease, dacryoadenitis, retinal vessels occlusions, retinopathy, maculopathy, endophthalmitis, cranial nerve palsies, optic neuritis, and uveitis with variable prognostic outcomes.^12,13^

The thought of children having more benign outcomes than adults is not completely accurate as health authorities alerted pediatricians about an outbreak of Kawasaki-like disease associated with clinical evidence of COVID-19.^14^ Children presented with a severe constellation of fever and multi-organ symptoms. Lo et al. reviewed more than 1400 patients diagnosed with MIS-C,^15^ the most common systemic manifestations were fever, gastrointestinal symptoms, shock, rash, and neurological symptoms. Patients were showing higher positivity to serology compared to nasopharyngeal swaps (80% vs 37%, respectively).^15^ Also, patients tended to present one month after their viral illness, implying a potential post-infectious inflammatory storm as the underlying mechanism.

Attention to ocular features may increase the sensitivity of case detection as one in ten cases may show at least one ocular manifestation.^11^ Generally, ocular manifestations may be underreported because studies focus on the consequences of the respiratory tract as COVID-19 may lead to fatal respiratory failure. Nonetheless, the literature still contains numerous reports that aid in outlining the ocular implications of this emerging entity in the pediatric population. This article aims to cover the various ocular manifestations in the literature of pediatric COVID-19 patients.

## Conjunctivitis

Conjunctivitis is an inflammation and swelling of conjunctival tissue with engorgement of blood vessels, epiphora, chemosis, ocular pain, follicular reaction of the tarsal conjunctiva, and occasional regional lymphadenopathy. Conjunctivitis is one of the potential initial symptoms of COVID-19 not only in adults but also in pediatric patients. The presence of conjunctivitis was linked in adult patients with severe disease compared to non-severe disease, while children did not manifest such an association with adverse disease course.^16^

Conjunctival manifestations can be found in newborns. A four-day-old baby presented with an acute onset of mucopurulent discharge with subconjunctival hemorrhage that was diagnosed as ophthalmia neonatorum.^17^ There were no other systemic manifestations. Although her parents tested negative before the mother’s admission for delivery, nasopharyngeal and conjunctival swabs were positive. Other investigations were noncontributory leading to attributing her ocular disease to COVID-19. Perez et al. in his cohort described 15 neonates who had COVID-19,^18^ eleven of them developed chemosis and hemorrhagic conjunctivitis. The author reasoned that such presentation may be due to an overlap between COVID-19 and co-morbidities of prematurity.

Wu et al. described a two-year-old boy who was diagnosed after a community screening test He was initially asymptomatic but later developed conjunctivitis and eyelid dermatitis that subsided five days later. They attributed this manifestation as either a direct infection of the virus or as a coinciding bacterial infection.^19^ Also, Dashti et al. reported conjunctivitis seven days after diagnosing a three-year-old child based on respiratory, systemic, and laboratory evidence of the infection.^20^ His ocular symptoms were alleviated after five days. Valente et al. reported four children with mild conjunctival inflammation, and clinical resolution was achieved after three to five days from onset.^21^ These cases demonstrate the possible acute occurrence of conjunctivitis in pediatric patients. Conjunctivitis was evident as a part of MIS-C in nearly half of the patients as described by Lo et al. and other reviews echoed similar findings^15,22–25^. Conjunctivitis in children is more likely induced by a systemic inflammatory reaction, specifically in the convalescent phase of the infection rather than a direct viral infection compared to adult patients.^26^

## Episcleritis

Episcleritis, inflammation of episcleral tissue and vessels, was described within the spectrum of manifestations in the literature in very few cases.^26^ It was evident in a 17-year-old boy who had an active illness with accompanying conjunctivitis, and another 13- year old boy presented in the convalescent stage where he had positive IgG immunoglobulins upon testing and developed optic neuritis a few days after recovery.^26^ Episcleritis was earlier described in two adult patients who developed this manifestation as an initial presentation and amid the viral illness^27–29^. Similarly to these cases, episcleritis was previously linked to respiratory viral illnesses, thus, caution is needed in such times given the temporal association of such presentation with COVID-19.^28^

## Keratitis

Corneal findings were found in few patients, although not as a sole finding but these cases add on the manifestations associated with COVID-19. Three patients suffered from severe corneal epitheliopathy that resolved after one week of treatment.^30^

## Orbital inflammatory disease

Orbital inflammatory disease (OID) accounts for up to 6% of orbital disease in general.^31^ OID's exact cause remains unknown and has a highly varying clinical features that may target any orbital tissue as lacrimal glands, muscles, or fat. Amid the pandemic, authors described the emergence of peculiar orbital manifestations in children. Eleiwa et al. described a case of orbital myositis in a child who was found to be positive COVID-19. The child presented with unilateral progressive periorbital swelling, eyelid drooping, left gaze horizontal diplopia, and painful ocular movements. Imaging supported the diagnosis, and steroids led to complete recovery. Orbital inflammation was also reported by Christopher and colleagues in a six-month-old infant who presented with periorbital edema that recurred.^32^ She had an active COVID-19 infection. Orbital biopsy showed comparable findings to lung specimens seen in patients with COVID-19, thus, diagnosing bilateral orbital inflammation presumably caused by COVID-19. Also, Remppis et al. reported in his case series an infant who presented with orbital swelling, fever, and other gastrointestinal features.^33^ Perez et al. described 15 neonates diagnosed with COVID-19.^18^ All cases exhibited periorbital edema, which may be attributed to either COVID-19 or prematurity as per the authors.

## Orbital cellulitis

Orbital cellulitis occurred in two patients who had sinusitis and intracranial abnormalities and co-infection with COVID-19. Turbin et al. described two adolescent patients who presented similarly in 24 h in two different emergency departments.^34^ They presented with unilateral progressive orbital swelling, restricted extraocular movement, proptosis. Extensive investigations were unremarkable. Despite the eventful hospital course, the outcome for the aforementioned cases was favorable.

## Retinal changes

Few patients have been reported to manifest retinal pathology attributed to COVID-19. Quintana–Castanedo et al. described unilateral pathological retinal findings in an 11-year-old child presenting with cutaneous features.^35^ Fundus examination showed retinal vascular changes, exudate, and perivascular infiltrate. Abbinante et al. also reported retinal changes that persisted during follow-up without any visual complaints.^36^ These changes were vascular tortuosity, especially arterial vasculature, and cotton wool spots along the vessels visible on examination and ancillary tests such as OCT and angio-OCT. Retinal vasculitis was discovered because of vigilance to the potential thromboembolic risk of COVID-19.

Posterior segment manifestations were associated with MIS-C in two children.^37^ An eight-year-old child presented complaining of floaters, with an otherwise unremarkable history. His examination showed splinter-retinal hemorrhages around the left optic nerve, and several vitritis-like hyperreflective dots in the right posterior vitreous on Optical Coherence Topography (OCT). Another child presented with conjunctival hyperemia and periorbital rash. Her examination revealed nonpurulent conjunctivitis, dilation and minimal tortuosity of retinal vessels, and vitritis-like hyperreflective dots in both eyes. Both patients had positive serology, and their ocular manifestations regressed during follow-up.

## Retinal vein occlusion

Walinjkar et al. described unilateral retinal vein thrombosis in a 17-year-old girl.^38^ She presented with diminishing vision. Her examination and ancillary tests supported the diagnosis of central retinal vein occlusion. She improved after receiving multiple intravitreal anti-Vascular Endothelial Growth Factor injections. This clinical presentation may be attributed to COVID-19, as retinal vessels occlusions are repetitively found in the literature in adults and seems to be one of the most common retinal presentations of COVID-19 in adults. Despite this, thromboembolic risk in pediatric patients may be lower. A recent multicenter cohort found that the incidence of thromboembolic events in COVID-19 infected hospitalized pediatric patients was 2%, in MIS-C was 6.5% and 0.7% in asymptomatic patients.^39^ Factors associated with thrombosis were age more than 12, presence of a central venous catheter, cancer, and MIS-C. The former three factors were already established in hospitalized pediatric patients.

## Uveitis

Uveitic manifestations were associated with MIS-C, and not as an isolated COVID-19 associated finding in pediatric patients. Anterior uveitis was reported in several cases as either an initial manifestation of the syndrome or days after diagnosis.^30,40,41^ Five patients with MIS-C were diagnosed with bilateral non-granulomatous anterior uveitis.^30^ Chung et al. reported a 12-year-old child who developed blurred vision while hospitalized as a case of multisystem inflammatory syndrome.^40^ Bilateral anterior uveitis was diagnosed based on mild anterior chamber reaction and conjunctival hyperemia that was successfully treated by topical steroid. Another child presented with redness and tearing of eyes among other constellations of symptoms.^41^ Ophthalmological examination revealed bilateral non-granulomatous anterior uveitis with few keratitic precipitates, anterior chamber cells, and bilateral disc edema. Therapeutic intravenous immunoglobulins was started, however, the patient developed a new-onset retro-orbital pain. She persisted to have ophthalmic complaints, raising inflammatory markers, and high IgG titer for COVID-19, thus, they commenced oral prednisone. She fully recovered three months later. Anterior uveitis was reported in an adult patient who succumbed from the multisystem inflammatory syndrome,^42^ supporting uveitis as a potential manifestation of this syndrome not only in children but also in adults .

## Optic nerve abnormalities

Pediatric optic neuritis is rare. Annual incidence reached 0.57 per one hundred thousand and increases with adolescents.^43^ Clinical variability is evident between pediatric and adult optic neuritis. Optic disc edema and bilateral involvement are observed more in children. Orbital pain is less frequent, but vision loss during an attack is more pronounced in children, however, recovery as regaining normal visual acuity is noticeably better.^43^ The exact pathophysiology behind the neurological manifestation in COVID-19 is uncertain. Discussed theories are: (1) direct invasion through hematogenous spread infecting endothelial cells of the blood-brain barrier through binding of spike protein and ACE2 receptor or direct access through the olfactory neurons and disseminates using retrograde axonal transport.^44^ (2) Post-infectious immune-mediated molecular mimicry inducing an autoimmune reaction in the body.^45^ (3) Indirect mechanism by the cytokine storm and overt inflammation leading to indirect viral disruption of the blood-brain barrier and pro-coagulable status.^46^

Optic neuritis was described as an initial presentation of COVID-19.^47^ A previously healthy ten-year-old girl was diagnosed with unilateral optic neuritis and found to have a positive COVID-19 swab despite being asymptomatic. Another case presented with subacute visual loss that progressed with near blindness over seven days.^48^ The patient was diagnosed with anti-Myelin Oligodendrocyte Glycoprotein positive bilateral optic neuritis attributed to presumed infection with COVID-19. He was exposed to COVID-19 and suffered a febrile illness. Also, a 13-year-old boy presented to the hospital with a new-onset blurry vision in his right eye.^49^ Imaging studies supported optic neuritis. Extensive investigations were unremarkable except for positive serology for COVID-19; thus, the patient was labeled with post-infectious optic neuritis. Appropriate treatment with steroids led to the disappearance of symptoms within three weeks. Additionally, two teenagers suffered from optic neuritis (bilateral and unilateral cases), which was temporally associated with COVID-19 clinically and serologically.^49^ Overall, the patients did not have any demyelinating findings on imaging or previous history of neurological disease. They had favorable visual outcomes concurring the known prognosis of pediatric optic neuritis, without any reportable relapse indicating ongoing inflammatory processes.

Multisystem inflammatory syndrome in children was associated with Idiopathic intracranial hypertension. It was shown to have manifested with ophthalmic features as reported by Verkuil et al..^50^ A 14-year-old nonfebrile girl presented with a headache and right abducent nerve palsy. Her examination revealed bilateral papilledema. Brain imaging was consistent with elevated intracranial pressure, and serology proved evidence of COVID-19 confirming the diagnosis. She recovered after receiving acetazolamide and oral steroids. Baccarella et al. reported two cases,^51^ the first case presented with diplopia and a worsening headache following few days of a febrile illness, clinical examination revealed unilateral abducent nerve palsy without papilledema. A lumbar puncture (LP) showed high opening pressure. He was treated with acetazolamide and discharged with improving clinical status. The other patient presented with diplopia and persistent headache after being discharged after hospitalization due to MIS-C. He had unilateral abducent nerve palsy with bilateral papilledema due to elevated intracranial pressure. Brain and orbits imaging supported the diagnosis, however, lumbar puncture showed normal opening pressure. Diplopia resolved the day after the LP, but papilledema persisted. He was discharged with steroids and showed resolution of his illness during follow-up.^51^

## Abnormal ocular motility

Other cranial nerves involvement has been rarely described in pediatric patients with confirmed COVID-19. A 15-year-old girl with serology confirmed COVID-19 was diagnosed with bilateral optic neuritis and unilateral abducent nerve palsy presenting as diplopia.^49^ A two-year-old girl presented with acute onset oculomotor nerve palsy.^52^ She tested positive for COVID-19 by a nasopharyngeal swap, although no systemic symptoms were apparent. Both cases recovered completely and partially, respectively. Another unique clinical case, albeit not a true cranial nerve involvement, was described in a four-month-old infant who developed chaotic ocular movements associated with tongue thrusting after one month from her COVID-19 illness.^53^ She did not exhibit developmental regression and completely recovered after corticosteroids. Several reports have described cranial nerves involvement in adults.^54–58^ Most cases had spontaneous regain of normal neurological function without deficits.

## Conclusion

**Figure 1.**
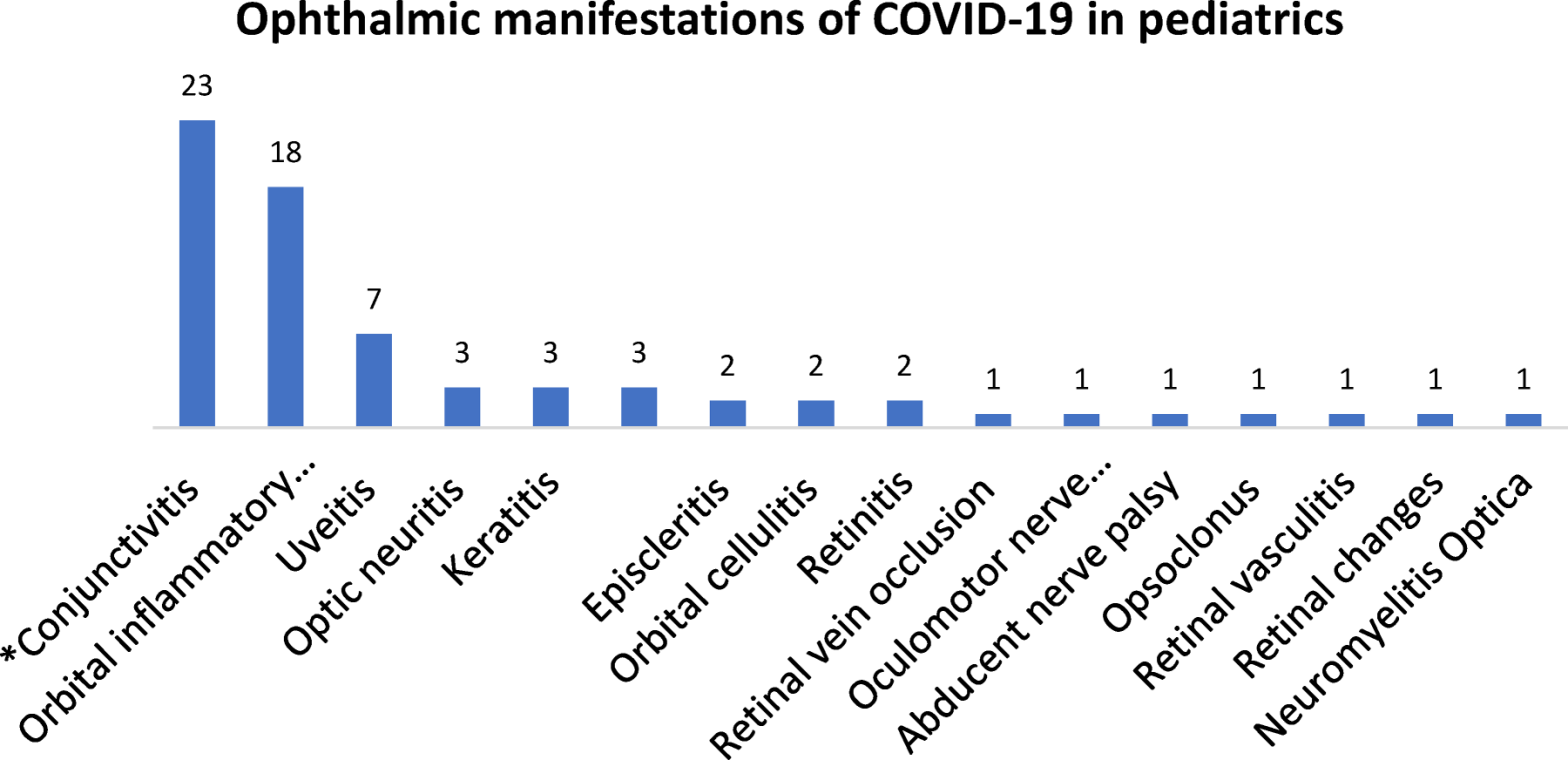
Reported ophthalmic manifestations in published case-reports and case-series of COVID-19 & MIS-C.

**Figure 2.**
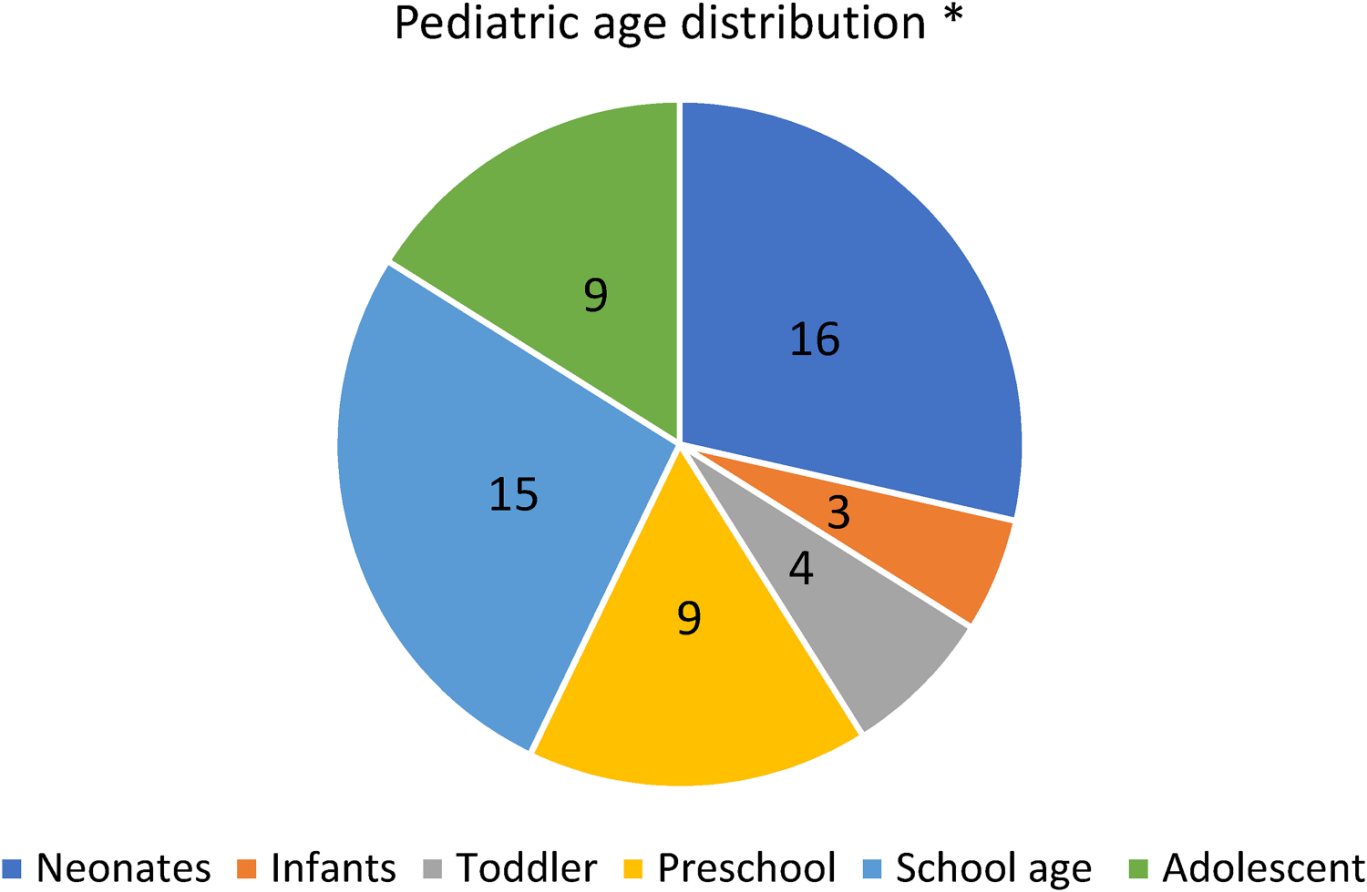
Age group distribution of patients with ophthalmic manifestation of COVID/MIS-C. (n = 56)

This review outlines the vast spectrum of ocular manifestations of COVID-19 in the pediatric age groups (Figures 1–2). Ocular manifestations maybe divided into two categories (Table 1): multisystem inflammatory syndrome related manifestations and COVID-19 related manifestations. The literature suggests that each has distinct pathophysiology. MIS-C may initially present with ocular manifestations; thus, ophthalmologists should be aware and have a high index of suspicion, specially when it presents with other systemic symptoms in order to promptly manage these cases interdisciplinary and limit potential deterioration in vision and health in general. The management is generally supportive and specific to each clinical manifestation. Ocular manifestations of MIS-C have a benign clinical course, and cases reported in the literature had uneventful visual recovery after receiving the treatment for each disease. The other isolated manifestations carried a similar benign outcome as patients regained any visual deficit and recovered to their normal status; however, the long-term prognosis is unknown just like MIS-C.

*Conjunctivitis reported in review articles of MIS-C were not included.

*Only patients in case reports and case-series were included.

**Table 1.** Summary of pediatric ocular manifestations attributed to COVID-19.

***MIS-C related manifestations***
Conjunctivitis
Anterior uveitis
Corneal epitheliopathy
Optic neuritis
Idiopathic intracranial hypertension
Retinitis
***COVID-19 related manifestations***
Conjunctivitis
Ophthalmia neonatorum
Optic neuritis
Abducent nerve palsy
Oculomotor nerve palsy
Opsoclonus
Neuromyelitis Optica
Orbital Inflammation Disease
Orbital cellulitis
Orbital myositis
Retinal vasculitis
Retinal vein occlusion
Asymptomatic retinal changes
